# Fluorescent Probe-Based Fiber Optic Sensor for Real-Time Monitoring of Chloride Ions in Coastal Concrete Structures

**DOI:** 10.3390/s24123700

**Published:** 2024-06-07

**Authors:** Zhen Lin, Quanfeng Ouyang, Chuanrui Guo, Yiqing Ni

**Affiliations:** 1 College of Civil and Transportation Engineering, Institute of Urban Smart Transportation & Safety Maintenance, Shenzhen University, Shenzhen 518060, China; zhenzz.lin@connect.polyu.hk (Z.L.); oyqfeng0218@163.com (Q.O.); 2 National Key Laboratory of Green and Long-Life Road Engineering in Extreme Environment (Shenzhen), Shenzhen 518060, China; 3 Shenzhen Key Laboratory of Safety and Health Monitoring of Marine Infrastructures, Shenzhen 518060, China; 4 Department of Civil and Environmental Engineering, The Hong Kong Polytechnic University, Hung Hom, Kowloon, Hong Kong SAR, China

**Keywords:** chloride ion monitoring, coastal concrete structure, fluorescent probe, fiber optic sensor, quinine sulfate

## Abstract

Coastal concrete structures, such as cross-sea bridges and tunnels, are susceptible to the penetration of chloride ions, which can lead to the deterioration of the passive film on the rebar surface, consequently accelerating the corrosion process. Conventional methods for monitoring chloride ions typically require in situ drilling for sample collection, thereby compromising efficiency and accuracy. Additionally, real-time monitoring and early warning cannot be achieved. To address these challenges, this work introduces a fluorescent-probe-based fiber optic sensor for monitoring chloride levels in concrete structures. Quinine sulfate was chosen as the fluorescent material due to its exceptional sensitivity to chloride ions and its stability in concrete environments. The proposed sensor was manufactured using sol–gel and 3D-printing techniques. Tests were conducted using concrete simulation fluid and cement mortar specimens. The results demonstrate that the sensitivity of the proposed sensor is greater than 0.01 M, and its accuracy in penetration depth measurement is better than 3 mm. The findings confirm that the designed fiber optic sensor based on quinine sulfate enables real-time monitoring of chloride ions in concrete structures, offering high sensitivity (0.1% in concentration and 2.7 mm in terms of penetration depth), unique selectivity (as it is immune to other ions whose concentrations are 10 times higher than those of Cl^−^), and a compact size (10 × 20 mm). These attributes render it promising for practical engineering applications.

## 1. Introduction

Coastal concrete infrastructures, including cross-sea bridges and tunnels, have witnessed significant growth in recent years. As these structures age, they become increasingly susceptible to chloride ion penetration from the marine environment. Such penetration can undermine the protective passive film on steel reinforcement, accelerate the corrosion process, diminish structural performance, and ultimately compromise the safety of a structure [1,2]. Chloride ions within concrete exist in two distinct forms: free chloride ions and those bound within hydration products [3,4]. It is the presence of free chloride ions that predominantly contributes to the corrosion of reinforcing bars. Monitoring the levels of free chloride ions in concrete serves as a vital early-warning system for rebar corrosion, thereby ensuring the safety and longevity of the structure.

The currently employed methods for monitoring chloride-induced erosion in concrete include titration, colorimetry, and ion-selective electrode techniques. The titration and colorimetric methods [5,6,7,8] require drilling or milling to collect sample powders, followed by chemical composition analysis using specific reagents in a laboratory. This process is time-consuming and only yields the total chloride ion content, encompassing both free ions and those bound in hydration products. The ion-selective electrode method [9] involves embedding selective electrodes within the concrete structure beforehand to evaluate steel bar corrosion via corrosion potential or polarization resistance [10,11]. However, this technique is prone to interference from other ions—known as the ion selectivity issue—and it also requires the enhancement of monitoring sensitivity.

In recent years, the deployment of fiber optic sensing technology for the structural monitoring of concrete has been notable, leveraging its compact size, high sensitivity, strong anti-interference capabilities, and resilience in challenging environments [3,8,12,13]. To date, fiber optic sensors have predominantly been used for post-corrosion monitoring, in which they are used to measure corrosion-induced strain or mass loss [14,15], which limits their utility for early corrosion warning. Even though long-period fiber grating (LPFG) has been employed to monitor chloride ion concentrations, it lacks ion-specific selectivity and only detects changes in the refractive index, leading to potential inaccuracies due to the presence of other ions such as sulfates and hydroxides and variations in pH [16].

Fiber optic fluorescent sensors employ optical fibers to transmit both excited and emitted fluorescence signals. These sensors, through the use of fluorescent substances, can attain highly sensitively detect various ions [13,17,18,19]. This technology provides a novel pathway for the monitoring of chloride ions in concrete. However, further investigation is required to reveal the optimal fluorescent substance, sensor fabrication procedure, and monitoring sensitivity to use in this regard.

This study introduces a novel approach in the form of a fiber optic fluorescence sensor designed for the detection of chloride ions in concrete, utilizing quinine sulfate as a fluorescence probe. The design and fabrication of the sensor are realized through a sol–gel process combined with 3D printing. We thoroughly investigated and evaluated the sensor’s sensitivity, accuracy with respect to concentration measurement, and penetration depth within both concrete simulation solution and cement mortar specimens.

## 2. Working Principle

The interaction between a fluorescent probe and analytes produces a fluorescence-quenching effect, where the intensity of the fluorescence peaks decreases with the increase in analyte concentration. The quenching process can be described using the Stern–Volmer equation [19]:

(1) $$\frac{I_{0}}{I}=1+K_{sv}[Q]$$

I_0_: Initial fluorescence intensity in the absence of a specific ion;
I: Fluorescence intensity in the presence of a specific ion;
K_SV_: Stern–Volmer constant;
[Q]: Ion concentration.

To produce fluorescence spectra under the irradiation of excitation light, it is crucial to select specific fluorescent indicators that can generate the quenching effect with the ions to be measured [20].

To compare the spectral responses of various indicators, Martin et al. [19] assessed the fluorescence spectra of both quinoline-type and acridine-type indicators under variable pH and chloride ion concentration conditions. The findings indicated a heightened sensitivity of quinoline-type indicators towards chloride ions compared to their acridine counterparts, with the capability of detecting chloride ion concentrations as low as 10 mM. Additionally, these indicators exhibited consistent fluorescence characteristics, rendering them particularly apt for the analysis of chloride ions within neutral to alkaline environments. Specifically, quinine sulfate was employed in Martin’s experiments as a quinoline-type chloride indicator, characterized by its robust physicochemical stability, effective solubility in aqueous solutions, dual excitation wavelengths at 250 nm and 350 nm, and emission peaks spanning 450–500 nm [21]. Consequently, quinine sulfate was the fluorescent indicator chosen for chloride ion concentration assessment in this study.

Sujatha Jayaraman [21] further explored the quenching behavior of quinine sulfate fluorescence upon interaction with chloride ions, ascertaining that the quenching effect is predominantly caused by collisional encounters with heavy atoms. The observed quenching mechanism, termed collision quenching (CQ), was attributed to the perturbative influence of halogen ions’ heavy atom effect on the fluorescence of the quinine-type sensor. Essentially, this effect can significantly diminish the fluorescence emitted by the quinoline sensor. Leveraging this fundamental understanding, this study has effectively employed a means of monitoring chloride ion concentrations.

## 3. Sensor Fabrication

Incorporating quinine sulfate in its powdered form into the design of a sensor requires a protective “encapsulation” process. Utilizing the inherent transparency, mechanical robustness, chemical stability, and flexibility of sol–gels, this study utilized sol–gel technology to embed quinine sulfate effectively within a solid matrix. The selected precursor solution comprised a volumetric ratio of tetraethyl orthosilicate (TEOS) to anhydrous ethanol and hydrochloric acid (0.01 M) of 4.9:8:1.6, respectively [22]. The quinine sulfate solution was prepared at a concentration of 4 × 10^−3^ M, supplemented with formamide at a volumetric ratio of 15%.

The sol–gel process was initiated with the preparation of a 5 × 10^−2^ M quinine sulfate solution, and then 2055 μL of tetraethyl orthosilicate (TEOS), 3285 μL of anhydrous ethanol, 500 μL of 0.01 M hydrochloric acid, 800 μL of 5 × 10^−2^ M quinine sulfate solution, and 1260 μL of formamide were poured into a beaker to configure the sol–gel solution. The solution was stirred in a magnetic stirrer for 2 h at room temperature. After 1 day of resting, the fluidity of the sol was weakened, and it was in a semi-gel state. The sol was spin-coated on a quartz wafer at 600 rpm for 30 s and then cured for 1 h at a temperature of 50 °C.

Since the total volume of the sol–gel solution was 10 mL, the hydrochloric acid solution was equivalent to a twentyfold dilution. Thus, the chlorine ions contained in the sol–gel solution could be ignored.

**Figure 1:**
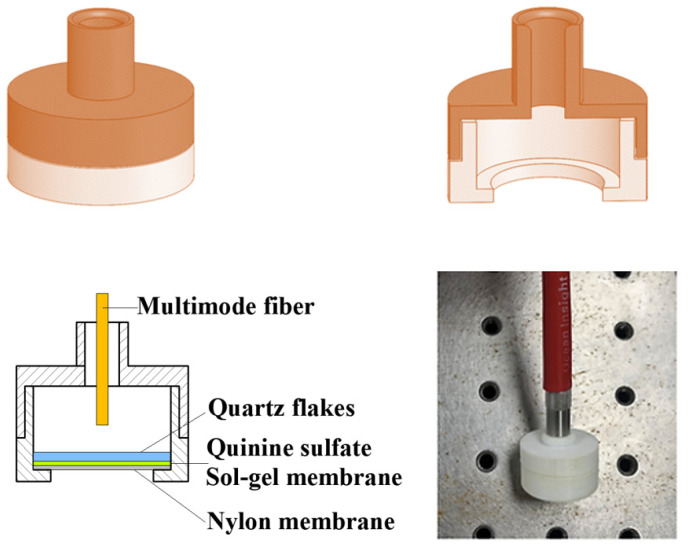
Sensor’s structural design.

As shown in Figure 1, the structure of the sensor is divided into two parts, with the bottom assembly consisting of sequentially stacked parts—a quartz sheet (1 mm in thickness and 10 mm in diameter), a quinine sulphate sol–gel membrane, and a nylon filter membrane (10 mm in diameter, with a 0.05 μm pore size)—that were securely bonded together with a waterproof adhesive. The upper housing of the structure was carefully designed to integrate seamlessly with multimode fiber connectors to ensure robust connectivity and signal integrity.

The upper and lower parts of the sensor were integrated with a fixed thickness of 8 mm and a fixed wall thickness of 2 mm, and different distances between the quartz sheet and the fiber optic tip were generated by varying the distance from the interface to the upper part of the housing. This distance was set to 1 mm, 2 mm, 3 mm, 4 mm, and 5 mm to investigate whether the distance between the quartz piece and the fiber optic tip affects sensitivity. Fluorescence tests were performed on the sensors at different distances, and the effect of this distance on sensitivity was measured using the ratio of fluorescence quenching: $I_{0}-I/I_{0}$. It was found that the highest sensitivity for the sensors was achieved at a distance of 2 mm. Therefore, the distance between the quartz sheet and the fiber optic tip was set to 2 mm in the sensor structure of this study.

**Figure 2:**
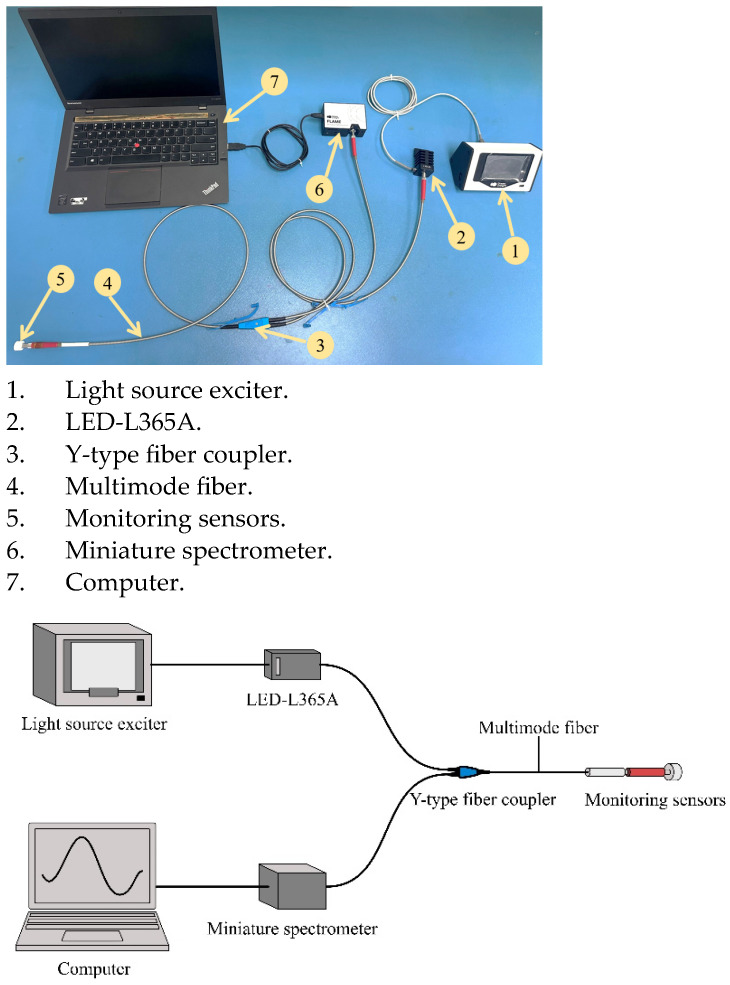
Fluorescence-monitoring system.

Figure 2 illustrates the comprehensive fluorescence monitoring system. The system’s operations are initiated by a light source exciter, specifically a current-driven LED module (Ocean Optics LED-365A) that generates 365 nm wavelength light. This light is transmitted through the upper branch of a Y-type optical fiber (Ocean Optics, Orlando, FL, USA, BIF200-UV-VIS, with a Fiber core size of 200 μm) and directed into a coupler, which then conveys the light to the fluorescent probe. Within the probe’s structure, the fluorescent indicator interacts with the chlorine ions present in the test liquid, resulting in a fluorescence-quenching reaction. Post-quenching, the modified fluorescent signal is captured by the Y-type optical fiber and subsequently relayed back through the coupler. After the coupler bifurcates the signal, the lower arm of the Y-type optical fiber channels the fluorescence signal into a spectrometer (Ocean Optics, Orlando, FL, USA, miniature spectrometer (FLAME-S, FLMS16325)). The spectrometer processes the signal, and the fluorescence intensity data are further analyzed and recorded using dedicated computer software, ensuring the information is preserved for future reference and analysis.

## 4. Experiments

### 4.1. Calibration

**Figure 3:**
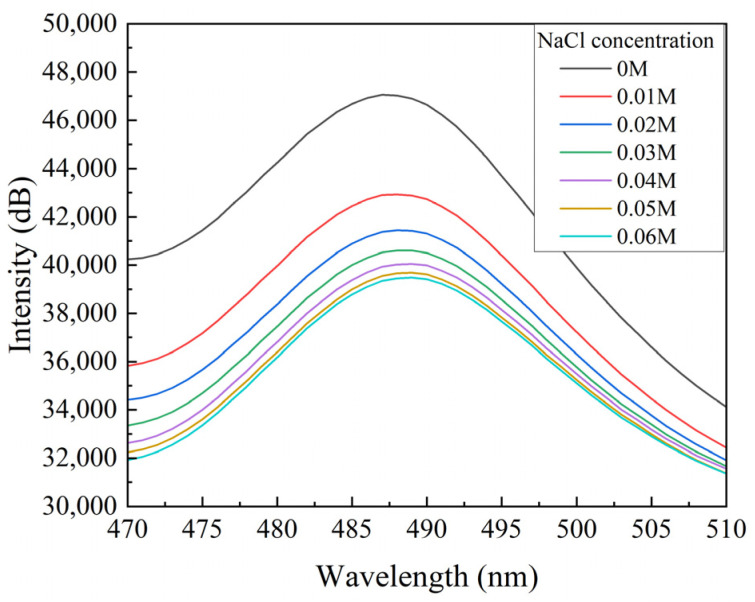
Fluorescent spectra of quinine sulfate under different NaCl concentrations.

The sensors were put into different concentrations of NaCl solution, and the concentration gradient was set to 0.01 M, with a starting concentration at 0 M. The fluorescence test was carried out separately to collect the fluorescence spectral information, as shown in Figure 3.

It can be seen that the fluorescence intensity of quinine sulfate decreases with the increase in chloride ion concentration even when the NaCl concentration is as low as 0.01 M. Due to the limited content of quinine sulfate in the sensitive membrane, the quenching of quinine sulfate tended to be saturated when the test concentration reached 0.07 M.

**Figure 4:**
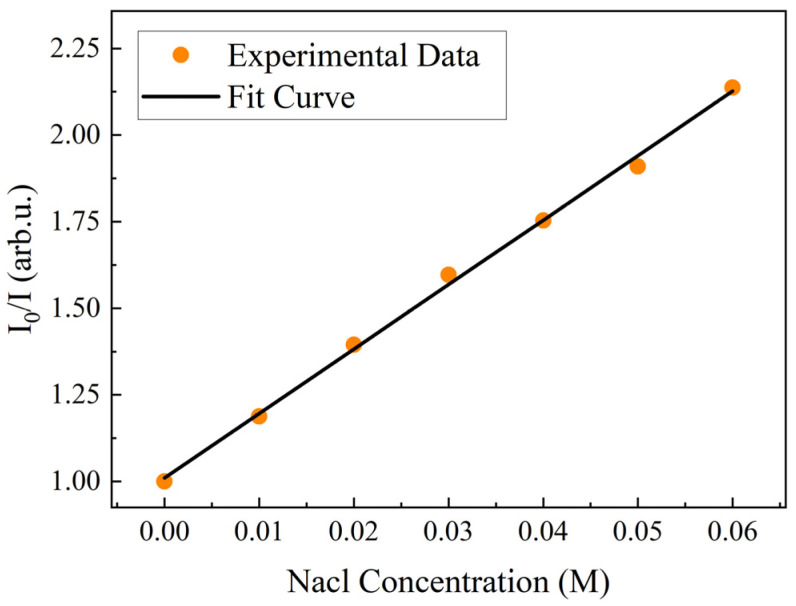
Calibration curve of chloride ion concentration versus fluorescence intensity.

By repeating the experiment five times and taking the average value, the calibration data were linearly fitted to the chloride concentration and fluorescence intensity according to the Stern–Volmer equation. Figure 4 shows the calibrated curve of quinine sulfate with respect to chloride ion concentration, and the corresponding calibration equation is

(2) $$\frac{I_{0}}{I}=1+18.609[{Cl}^{-}]$$

### 4.2. Test

In order to verify the performance of the sensors in concrete, the concrete simulation solution test and the resistance-to-ionic-interference test were carried out, and then the sensors were buried in mortar specimens to monitor the chloride ion concentration.

**Figure 5:**
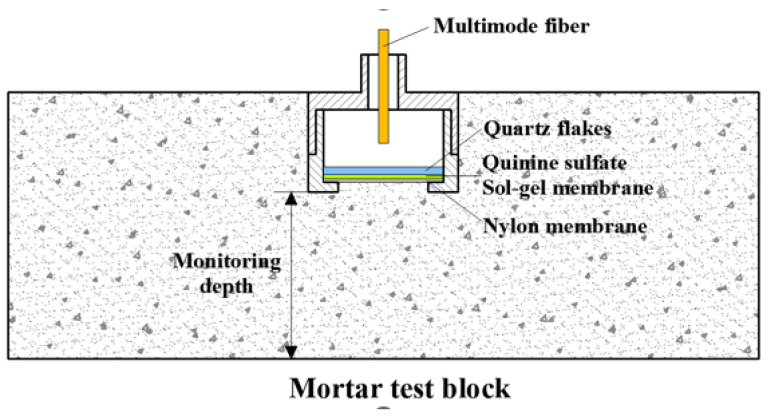
Schematic diagram of a sensor’s burial inside concrete.

**Figure 6:**
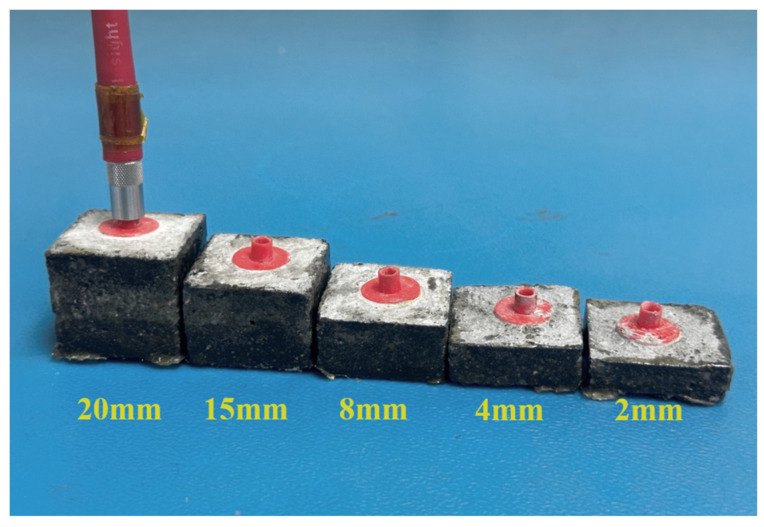
Sensors at different burial depths.

**Figure 7:**
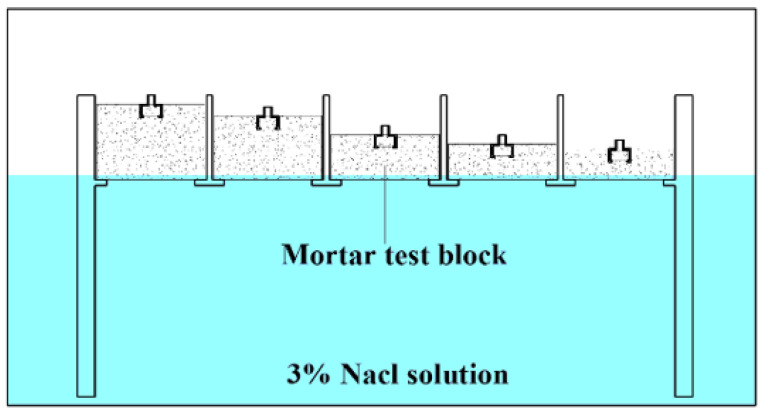
Experimental setup for the mortar specimen.

(a) Concrete simulation solution test: This study used Ca(OH)_2_ powder and a NaHCO_3_ standard solution to configure concrete simulation solutions with different pH values. The configuration process started with adding excess Ca(OH)_2_ powder to deionized water to obtain a supersaturated Ca(OH)_2_ solution. The supersaturated solution was left to stand for more than 24 h, and then the upper layer of the supersaturated solution was extracted, serving as the base of the concrete simulation solution. At this time, the pH value of the saturated Ca(OH)_2_ solution was 12.6, and the pH value of the simulation solution was adjusted by adding NaHCO_3_ at a concentration of 1 M drop by drop to the saturated solution, and thus concrete simulation solutions with pH values of 11.6, 10.6, 9.6, and 8.6 were obtained. The concentrations of chloride ions in the simulated concrete solutions were then adjusted using a known amount of NaCl, and the tests were carried out separately.
(b) Resistance-to-ionic-interference test: Ionic selectivity is the main index used to evaluate the performance of fluorescence sensors. The internal environment of a concrete structure is complex, and other ions may interfere with the detection of chloride ions. In this study, several anions, namely, ${SO}_{4}^{2-}$, ${NO}_{3}^{-}$, ${HPO}_{4}^{2-}$, ${CO}_{3}^{2-}$, and ${SiO}_{3}^{2-},$ and cations, namely, ${Na}^{+}$, ${Ca}^{2+}$, and ${Mg}^{2+}$, which are commonly found inside concrete, were selected to verify the ionic selectivity of the quinine-sulfate-based chloride ion sensor. The ratio of fluorescence burst, that is, $I_{0}-I/I_{0}$, was used to define the degree of influence of the added ions on the fluorescence intensity of quinine sulfate, and a control experiment with a blank group was conducted using deionized water, taking into account the dilution effect during the addition of the standard solution of each ion. The concentrations of the interfering ions were all 100 μg/mL, and the concentration of chloride ions was 10 μg/mL.
(c) Chloride ion concentration monitoring in cement mortar specimens: Mortar specimens with buried sensors (Figure 5) located at different depths (2 mm, 4 mm, 8 mm, 15 mm, 20 mm) and water/cement ratios (0.3, 0.4, 0.5) were prepared, with the specimens being 30 mm long by 30 mm wide. Control groups with no sensors were also prepared for chloride measurement using a conventional method. All specimens were subjected to standard curing for 28 days to ensure that the specimens acquired the expected strength, and Figure 6 shows the mortar specimens after demolding. The sides of all the specimens were sealed with epoxy resin to allow unidirectional penetration of the chloride ions. Figure 7 shows the overall experimental design. The dimensions of the squares in which the mortar specimens were placed were 32 mm × 32 mm, and all the specimens were placed in the same airtight chloride-salt environment at a constant temperature of 25 °C. The sodium chloride concentration was set to 3%, and the bottom of the specimen block was placed in contact with the solution and periodically replenished with a 3% sodium chloride solution.

The test period was set to 30 days, and the fluorescence intensity data of all the test blocks were recorded via uninterrupted testing in the first seven days of the test. Seven days later, the fluorescence intensity data on the 10th, 15th, 20th, 25th, and 30th days were recorded.

The control groups were treated with potentiometric titration to determine the chloride ion concentrations on days 10, 20, and 30. The powder was ground layer by layer starting along the eroded end face of the test side. Powder samples corresponding to different depths, i.e., 2~3, 4~5, 8~9, 15~16, and 20~21 mm, were taken for further analysis. Chloride ion content w (by mass of concrete) was determined based on AASHTO T260-97 (2009) “Standard method of test for sampling and testing for chloride ion in concrete and concrete raw materials”.

### 4.3. Results and Analysis

In order to facilitate comprehension of the results and analyze them in comparison with the control group, the concentration of chloride ions (M) in the concrete was converted to percentage of chloride content (%) according to the calibration equation.

#### 4.3.1. Concrete Simulation Solution Test

**Figure 8:**
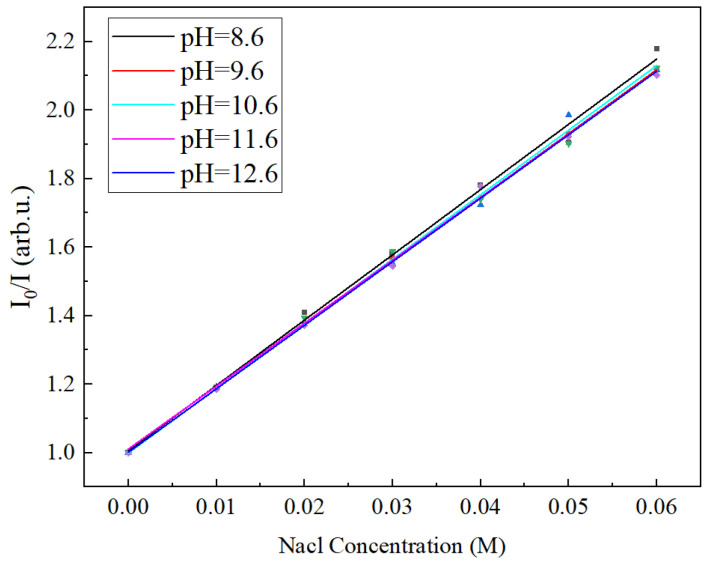
Calibration of quinine sulfate with respect to chloride ion concentration in different-pH concrete simulants.

The test data were linearly fitted with different chloride concentrations and fluorescence intensities according to the Stern–Volmer equation. Figure 8 shows the K value and standard curve of the sensor with respect to different chloride ion concentrations in the concrete simulant at different pH values. The standard equations at each pH are as follows:

(3) $$I_{0}/I=1+19.049[{Cl}^{-}];\;\mathrm{pH}=8.6\;I_{0}/I=1+18.61[{Cl}^{-}];\;\mathrm{pH}=9.6\;I_{0}/I=1+18.842[{Cl}^{-}];\;\mathrm{pH}=10.6\;I_{0}/I=1+18.375[{Cl}^{-}];\;\mathrm{pH}=11.6\;I_{0}/I=1+18.531[{Cl}^{-}];\;\mathrm{pH}=12.6$$

It can be seen that the sensor performed stably in an alkaline concrete simulation solution environment with a pH range of 8.6 to 12.6. The sensitivity of the sensor (sensitivity k-value) fluctuated within a very small error range and was essentially the same as the sensitivity k-value of 18.609 observed during the initial calibration. Therefore, this quinine-sulfate-based fiber optic chloride sensor can monitor chloride in different alkaline concrete environments, and an environment’s pH value has little effect on the sensor’s performance.

#### 4.3.2. Ion Resistance Test

**Figure 9:**
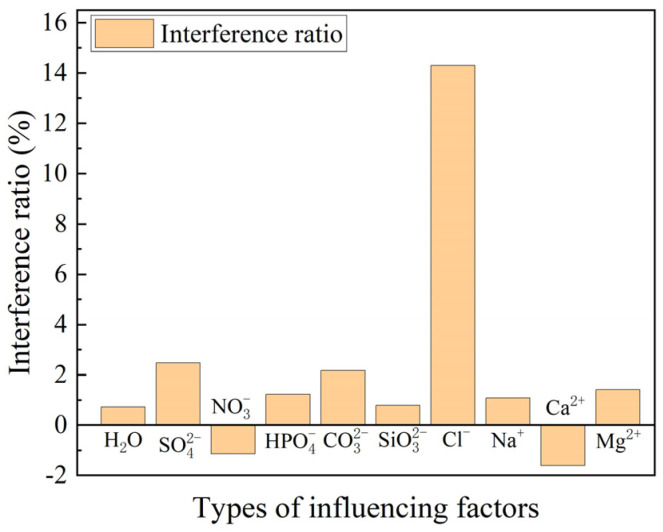
Resistance to ionic interference.

Figure 9 shows the ion resistance test results for quinine sulfate. The fluorescence burst ratios of ${SO}_{4}^{2-}$, ${NO}_{3}^{-}$, ${HPO}_{4}^{2-}$, ${CO}_{3}^{2-}$, ${SiO}_{3}^{2-}$, ${Na}^{+}$, ${Ca}^{2+},$ and ${Mg}^{2+}$ were 0.0247, 0.0114, 0.0123, 0.0217, 0.0079, 0.0108, −0.0161, 0.0141, and 0.0072 for the blank control group, consisting of deionized water, and these values are much smaller than the fluorescence burst ratio of 0.1413 for ${Cl}^{-}$. Considering the dilution of the fluorescent complex by the solvent in the blank group (consisting of deionized water) resulted in reduced fluorescence with a burst ratio of 0.0072, and the concentrations of the above-referenced ions in the experiments were all set to 10 times the concentration of ${Cl}^{-}$. Therefore, the results showed that ${SO}_{4}^{2-}$, ${NO}_{3}^{-}$, ${HPO}_{4}^{2-}$, ${CO}_{3}^{2-}$, ${SiO}_{3}^{2-}$, ${Na}^{+}$, ${Ca}^{2+}$, and ${Mg}^{2+}$ have almost no effect on the bursting of quinine sulfate by ${Cl}^{-}$. Our fiber optic sensor based on quinine sulfate has good resistance to ionic interference and strong selectivity for chloride ions.

#### 4.3.3. Mortar Specimen Test

**Figure 10:**
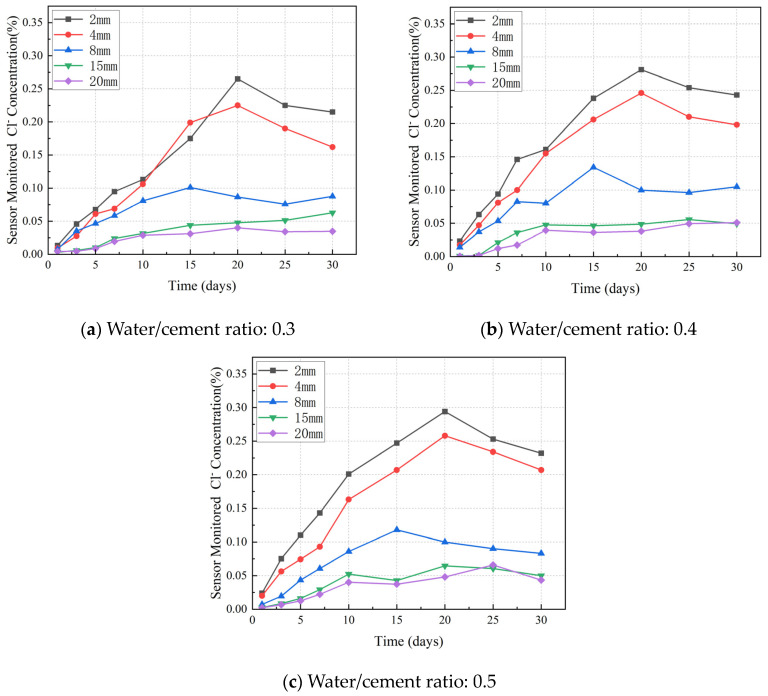
Sensor-monitored chloride concentrations for different w/c ratio specimens.

Figure 10a presents the data monitored by the sensor at various depths: 2 mm, 4 mm, 8 mm, 15 mm, and 20 mm. It was observed that the penetration rate was faster at the depths of 2 mm and 4 mm compared with the others. Until the 20th day, the chloride concentration was significantly reduced due to its binding effect with respect to the aqueous compounds inside the concrete.

Figure 10a–c also indicate that an increase in the water/cement ratio of the specimen correlates with a rise in the concentration of chloride ions at equivalent depths. The smaller the water/cement ratio, the smaller the maximum geometric pore size and critical pore size of the concrete, the higher the degree of refinement of the pore structure, and the better the resistance to chlorine ion permeability.

**Figure 11:**
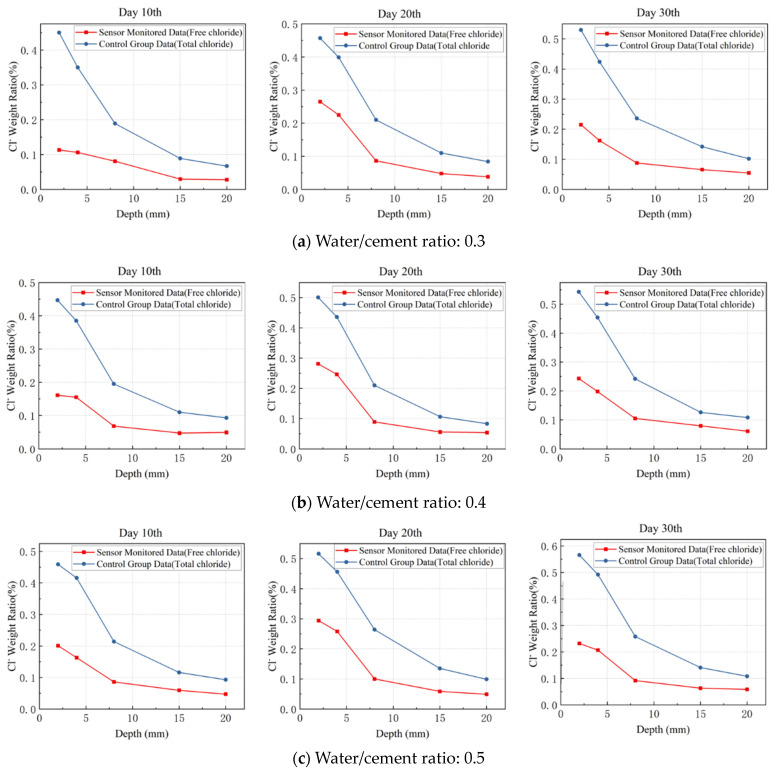
Chloride-ion-concentration-monitoring data vs. control data.

The data obtained from the control group are the total chloride ion concentrations determined using the potentiometric titration method, while the sensor monitored the free chloride ion concentration. Free chloride ions will combine with the hydration products in concrete during infiltration, and as infiltration occurs, the free chloride ion concentration within the concrete will be lower than the total chloride ion concentration. Figure 11a–c shows the sensor-monitored chloride concentrations and total chloride concentration for each water/cement ratio. The chloride concentration derived from the sensor (free chloride) is generally smaller than that derived from the potentiometric titration method (total chloride concentration), which verifies the above illustration.

The difference between the free and total chloride ion concentrations exhibits a decline with an increasing depth. This phenomenon can be attributed to the higher chloride ion concentrations at shallower depths, allowing a greater number of chloride ions to interact with and bind to available sites within the concrete matrix. This elevated interaction and binding capacity at superficial levels results in a pronounced difference between the free and total chloride ion concentrations. Conversely, as depth increases, the concentration of chloride ions diminishes, leading to a corresponding decrease in their binding strength. Consequently, the differential between free and total chloride ion concentrations becomes less marked. This gradient underscores the dynamic nature of chloride ion interactions within the concrete matrix, where the concentration and binding strength of chloride ions play pivotal roles in defining the observed concentration profiles at varying depths.

Penetration depth measurement accuracy: The response time of each depth sensor was taken as the response time (the point at which the sensor sensed the presence of chloride ions), while the actual depth of chloride erosion was determined by using potentiometric titration for different depths of the same test block. Taking the monitoring depth of 2 mm as an example, samples were obtained from the 1–2, 2–3, and 3–4 mm depths of the specimen blocks in layers, and the samples were processed according to AASHTO standard [23] to determine the chloride content and to derive the concentration of chloride ions at a depth that is similar to the sensor’s response concentration. Depths similar to the response concentration of the sensor were taken to be the actual depths of penetration, and the average value of the result was taken to be the reference value *d*_0_. The sensor test and potentiometric titration test were each performed three times to allow us to take the average value, and the difference between the sensor’s test value, *d_t_*, and the reference value, *d*_0_, was defined as the accuracy of the depth of penetration monitoring, *P_d_*. The results in Table 1 show that the sensor’s accuracy was better than 3 mm.

Chloride concentration measurement accuracy: To compare the results for the proposed sensor and the potentiometric titration, it was necessary to transform the total Cl^−^ ions to free Cl^−^ ions since the sensor monitors free Cl^−^ while potentiometric titration measures total Cl^−^. The free chloride content was calculated to be between 10% and 25% of the total Cl^−^. Although the estimate of 10% may appear weak, in the presence of mortar samples, where the cement concentration is high in terms of the total weight of the samples, a greater proliferation of connections for bound chloride is possible. The other limit of 25% pertains to previous experimental data [3]. Therefore, 25% of the total chloride content was taken as the reference value, and the absolute value of the difference between the sensor test value and the reference value was taken as the sensor’s erosion-concentration-sensing accuracy. Taking the results on day 30 as an example, the potentiometric titration test value (the average of three test values) was used as the reference value *C*_0_, and the difference between the sensor test value *C_t_* (the average of three test values) and the reference value *C*_0_ was defined as the penetration-depth-monitoring accuracy *P_c_*. Table 2a–c shows the monitoring results of the mortar specimen with different water/cement ratios. It is shown that the sensing accuracy for chloride concentration is better than 0.1%.

## 5. Conclusions

In this study, we introduce a novel fiber optic fluorescent chloride ion sensor, utilizing quinine sulfate as the active sensing element. This study comprehensively evaluated this sensor’s performance through a series of designed experiments. These included testing it in simulated concrete solutions to assess its operational stability and resistance to various ionic interferences. The sensor’s efficacy in monitoring chloride ion concentrations was rigorously examined by embedding it at various depths within cement mortar specimens. The results allow several conclusions to be drawn on the sensor’s capabilities and potential applications in the field of concrete structural health monitoring:

(1) Fluorescence-based chloride ion sensing: Quinine sulfate exhibits robust fluorescence properties, wherein the fluorescence intensity proportionally correlates with the concentration of free chloride ions in accordance with the Stern–Volmer law. This relationship is crucial for sensor calibration. The experimental results demonstrate that the fiber optic fluorescent chloride ion sensor, utilizing quinine sulfate as a fluorescent agent, achieved a sensitivity exceeding 0.01 M. Such sensitivity proves this sensor’s capacity for precise chloride ion detection.
(2) Sensor stability and selectivity: The sensor maintains consistent performance across a range of pH values typically found in concrete simulation solutions, demonstrating minimal deviation in chloride ion detection. This stability, coupled with its resistance to ionic interference, highlights the sensor’s robustness. Furthermore, the sensor exhibits a superior selectivity for chloride ions, a critical attribute for reliable sensing in complex concrete environments.
(3) Chloride penetration-depth-sensing accuracy: The experimental results derived from the cement mortar specimens indicate that the sensor’s depth-sensing accuracy surpasses 3 mm. Notably, as the depth increases, the sensor’s concentration-sensing accuracy improves, achieving an overall accuracy greater than 0.1%. Remarkably, beyond a depth of 15 mm, the sensing accuracy enhances tenfold, achieving a precision better than 0.02%. However, it was observed that an increase in the water/cement ratio corresponds to a gradual decrease in sensing accuracy.

Future research endeavors will focus on investigating the impact of carbonization on the erosion rate of chloride ions, as well as on the performance and effective monitoring range of the sensor. A critical aspect of this research will involve quantifying the relationship between the depth of chloride ion erosion and its concentration, particularly distinguishing between carbonized and non-carbonized environments. Such investigations are imperative for enhancing the sensor’s applicability and accuracy in varied real-world conditions.

## Figures and Tables

**Table 1:** Chloride ion erosion-depth-sensing accuracy.

$d_{t}$ (mm)	$d_{0}$ (mm)	$P_{d}=\left|d_{t}-d_{0}\right|$ (mm)
2	3.3	1.3
4	3.3	0.7
8	6.3	1.7
15	16.7	1.7
20	17.3	2.7

**Table 2:** Perceived accuracy of chloride ion erosion concentration monitoring.

**(a) Water-to-Cement Ratio: 0.3**			
**Depths** **(mm)**	$C_{t}$ **(%)**	**25%** $C_{0}$ **(%)**	$P_{c}=\left|C_{t}-{25\%C}_{0}\right|$ **(%)**
2	0.22	0.13	0.09
4	0.16	0.11	0.05
8	0.09	0.06	0.03
15	0.07	0.04	0.03
20	0.06	0.03	0.03
**(b) Water-to-Cement Ratio: 0.4**			
**Depths** **(mm)**	$C_{t}$ **(%)**	**25%** $C_{0}$ **(%)**	$P_{c}=\left|C_{t}-{25\%C}_{0}\right|$ **(%)**
2	0.24	0.14	0.10
4	0.21	0.11	0.10
8	0.10	0.06	0.04
15	0.06	0.03	0.03
20	0.06	0.03	0.03
**(c) Water-to-Cement Ratio: 0.5**			
**Depths** **(mm)**	$C_{t}$ **(%)**	**25%** $C_{0}$ **(%)**	$P_{c}=\left|C_{t}-{25\%C}_{0}\right|$ **(%)**
2	0.23	0.14	0.09
4	0.20	0.12	0.08
8	0.11	0.07	0.04
15	0.08	0.04	0.04
20	0.06	0.03	0.03

## Data Availability

The raw data supporting the conclusions of this article will be made available by the authors on request.
